# Self-Reported Overall Adherence and Correct Inhalation Technique Discordance in Chronic Obstructive Pulmonary Disease Population

**DOI:** 10.3389/fphar.2022.860270

**Published:** 2022-08-12

**Authors:** Tereza Hendrychova, Michal Svoboda, Josef Maly, Jiri Vlcek, Eva Zimcikova, Tomas Dvorak, Jaromir Zatloukal, Eva Volakova, Marek Plutinsky, Kristian Brat, Patrice Popelkova, Michal Kopecky, Barbora Novotna, Vladimir Koblizek

**Affiliations:** ^1^ Department of Social and Clinical Pharmacy, Faculty of Pharmacy in Hradec Kralove, Charles University, Hradec Kralove, Czechia; ^2^ Institute of Biostatistics and Analyses, Ltd. Spin-off Company of the Masaryk University, Brno, Czechia; ^3^ Pulmonary Department, Klaudian Hospital, Mlada Boleslav, Czechia; ^4^ Department of Respiratory Diseases and Tuberculosis, University Hospital Olomouc, Olomouc, Czechia; ^5^ Faculty of Medicine, Palacky University Olomouc, Olomouc, Czechia; ^6^ Department of Respiratory Diseases, University Hospital Brno, Brno, Czechia; ^7^ Faculty of Medicine, Masaryk University, Brno, Czechia; ^8^ International Clinical Research Center, St. Anne´s University Hospital, Brno, Czechia; ^9^ Department of Pulmonary Diseases and Tuberculosis, University Hospital Ostrava, Ostrava, Czechia; ^10^ Faculty of Medicine in Ostrava, Ostrava, Czechia; ^11^ Department of Pneumology, University Hospital Hradec Kralove, Hradec Kralove, Czechia; ^12^ Faculty of Medicine in Hradec Kralove, Charles University, Hradec Kralove, Czechia; ^13^ Department of Pneumology, Bulovka Hospital, Prague, Czechia; ^14^ 3rd Faculty of Medicine, Charles University, Prague, Czechia

**Keywords:** COPD, adherence, compliance, application technique, inhalation systems

## Abstract

**Background:** Adherence to inhaled medication constitutes a major problem in patients with chronic obstructive pulmonary disease (COPD) globally. However, large studies evaluating adherence in its entirety and capturing a large variety of potentially associated factors are still lacking.

**Objective:** To study both elementary types of adherence to chronic inhaled COPD medication in “real-life” COPD patients and to assess relationships with a wide-ranging spectrum of clinical parameters.

**Methods:** Data from the Czech Multicentre Research Database (CMRD) of COPD, an observational prospective study, were used. Overall adherence (OA) was evaluated with Morisky Medication Adherence Scale (©MMAS-4) and adherence to an application technique (A-ApplT) with the Five Steps Assessment. Mann–Whitney *U* test, Spearman’s correlation, and logistic regression were used to explore relationships between variables.

**Results:** Data of 546 participants (69.6% of all patients from the CMRD) were analyzed. Two-thirds self-reported optimal OA, but only less than one-third demonstrated A-ApplT without any error. OA did not correlate with A-ApplT. Next, better OA was associated with higher education, a higher number of inhalers, a lower rate of exacerbations, poorer lung function, higher degree of upper respiratory tract symptoms (SNOT-22), absence of depressive symptoms, ex-smoking status, regular mouthwash after inhaled corticosteroids (ICS), and flu vaccination. By contrast, better A-ApplT was associated with a lower number of inhalers, better lung function, and regular mouthwash after ICS. Independent predictors of nonoptimal OA included lower degree of education, absence of flu vaccination, anemia, depression, and peptic ulcer history, whereas independent predictors of lower A-ApplT were lower education, absence of regular mouthwash after ICS, and higher COPD Assessment Test score.

**Conclusions:** Parameters associated with OA and A-ApplT differ, and those associated with both adherence domains are sometimes associated inversely. Based on this finding, we understand these as two separate constructs with an overlap.

**Clinical Trial Registration:** clinicaltrials.gov, identifier NCT01923051

## Introduction

Chronic obstructive pulmonary disease (COPD) represents a serious, primarily respiratory condition characterized by persistent multicomponent health and social difficulties, progressive airflow limitation, muscle dysfunction, and concomitant involvement of many organs (Global Strategy for the Diagnosis Management and Prevention of COPD, 2021).

Published studies from high-income countries reported a large variation in the prevalence of COPD. Prevalence estimates in the United States range from 10.2 to 20.9% (Ho et al., 2019). Researchers reported a similar variation in COPD diagnosis by a treating physician across Europe in several publications: 2.8% in Italy (Cazzola et al., 2011), 12% in Sweden (Danielsson et al., 2012), and 6.2% in a survey conducted in several European cities (Boutin-Forzano et al., 2007). In total, 711,000 individuals (6.7% of the entire population of the Czech Republic, EU) have been diagnosed with COPD at some point in their life (Zatloukal et al., 2020). In the Czech Republic, population-based life expectancy in COPD patients increased significantly from 2012 to 2018 (Koblizek et al., 2022; Valipour et al., 2022).

Even though COPD is not curable, the available pharmacological and nonpharmacological treatment modalities can help relieve symptoms and alleviate disease impact. Inhalation drugs are an essential component of pharmacological treatment (GOLD, 2021). As clearly demonstrated in the literature, patient adherence is crucial for the best possible effect of COPD treatment (Global Strategy for the Diagnosis Management and Prevention of COPD, 2021; Rogliani et al., 2017). On the contrary, substantial evidence exists that adherence to COPD treatment is poor (Bourbeau and Bartlett, 2008; Restrepo et al., 2008; Rogliani et al., 2017). This is why an increased emphasis is found on research in adherence, in COPD, and in general (Sanduzzi et al., 2014). A thorough knowledge of adherence among our patients enables us to work on addressing issues that may prevent our patients from adhering to their treatment.

For research, as well as clinical practice, we need to distinguish various aspects (types) of adherence. In respiratory medicine, we can distinguish between “quantitative” (e.g., proportion of doses used) and “qualitative” (e.g., handling of an inhalation device) adherence, noting that both concepts are equally important. In the present study, quantitative aspects of adherence are grouped under the term overall adherence (OA), whereas qualitative aspects are termed adherence to application technique (A-ApplT), often also called inhalation adherence (Vytrisalova et al., 2019). Each type of adherence requires a different approach to its measurement, interpretation, and consecutive patient management aimed at addressing the particular type of adherence.

In the field of COPD, studies of inhalation adherence predominate, although research focusing on OA also exists (de Oca et al., 2017; Jarab and Mukattash, 2019). Studies evaluating the relationships between the different aspects of adherence, especially application technique and incorrect dosing (forgetting, overmedication, doses omission, etc.), are still lacking.

For routine clinical practice, where time, human, and economic resources are very limited, demonstrating an easy and inexpensive approach to assess adherence is fundamental in order to identify individuals at high risk of nonadherence, e.g., based on easy-to-obtain demographic or clinical characteristics. Although “global” patient characteristics do not predict adherence well (Farmer, 1999), and even though it is generally believed that basic demographic variables (such as gender, age, educational level) may not be associated with adherence, some studies showed an association between adherence and these factors in patients with COPD (Bourbeau and Bartlett, 2008; Makela et al., 2013; Rogliani et al., 2017). Furthermore, studies of factors potentially associated with insufficient adherence, ideally in well-defined populations of patients with a particular disease, are needed.

Nowadays, no studies exist evaluating the broad spectrum of potentially relevant questions of OA and A-ApplT. The available data show that poor adherence in patients with COPD is associated with the complexity of medication regimens (often more than two different inhalation systems), lack of motivation, and psychiatric comorbidities (Bourbeau and Bartlett, 2008; Restrepo et al., 2008; Rogliani et al., 2017).

However, differences were observed between the “real-life” experiences and results from well-controlled clinical trials (Boudes, 1998; Vrijens et al., 2016).

This analysis aimed to assess adherence to chronic inhaled medication in patients with moderate to very severe COPD (non-mild COPD) in routine clinical practice. We focused on the following:

1) Overall adherence (OA) to COPD medication and its associated factors,
2) Adherence to application technique (A-ApplT) and its associated factors, and
3) Potential relationship between these two aspects of adherence.

## Materials and Methods

### Design and Participants

The present analysis used a baseline evaluation of medication adherence (OA) and adherence to application technique (A-ApplT) within the Czech Multicentre Research Database (CMRD) of COPD (Novotna et al., 2014; Czech Multicenter Research Database of COPD, 2022). CMRD was an observational long-term prospective multicenter study with the primary objective to investigate all-cause mortality in consecutive adult patients with non-mild COPD (postbronchodilator FEV_1_ ≤ 60% of predicted values) in the Czech Republic, EU. The study is registered at ClinicalTrials.gov NCT01923051. The design and elementary results from A-ApplT assessment have been published elsewhere (Vytrisalova et al., 2019). The study protocol was reviewed and approved by the Multicentre Ethics Committee, University Hospital Brno, the Czech Republic–EU (approval date 16 Jan 2013).

CMRD of COPD participants were recruited from 13 secondary and tertiary care pneumology centers providing specialized respiratory care to patients with COPD from August 2013 to December 2016. Written informed consent was obtained from all participants. The inclusion and exclusion criteria were described in detail previously (Novotna et al., 2014).

Evaluation of medication adherence was recommended as an important (but not mandatory) part of CMRD of COPD assessments (Novotna et al., 2014). Physical examinations, medical records, self-administered instruments, and interviews with patients were used to collect sociodemographic and health characteristics. Evaluation of potentially associated factors was based on the following self-administered instruments: Beck Depression Inventory Short Form (Beck Scale), Zung Self-Rating Depression Scale (Zung Scale), COPD Assessment Test (CAT), and the Sino-Nasal Outcome Test (SNOT-22) (Beck et al., 1961; Zung, 1965; Biggs et al., 1978; Jones et al., 1992; Furlanetto et al., 2005; Hopkins et al., 2009; Jones et al., 2009).

### Outcome Measures

Assessment of OA to chronic COPD medication was based on Morisky Medication Adherence Scale (©MMAS-4) (Morisky et al., 1986; Morisky et al., 1990; Morisky and DiMatteo, 2011). The ©MMAS-4 is a 4-item easy-to-use questionnaire with response alternatives yes (1 point) and no (0 points). The items are summed to give a range of scores from the lowest (4 points) to highest (0 points) adherence. A validated Czech version of ©MMAS-4 was used. The ©MMAS-4 was completed by a nurse based on participant responses. “©MMAS-4 test was used according to the license agreement concluded on 7 February 2013 between Charles University, Ovocný trh 560/5, 116 36 Praha 1—Staré Město, Faculty of Pharmacy in Hradec Králové, Ak. Heyrovského 1203/8, 500 05 Hradec Králové, Czechia and Donald Morisky, MMAS Research LLC, 14725 NE 20th, St. Bellevue, Washington 98007, as well as related documents, that also enabled the results from the COPD Study from the period of 7 February 2013 through 7 October 2018 to be freely used and published.”

Adherence to application technique (A-ApplT) was based on the Five Steps Assessment used for the first time and described in detail in our previous publication (Vytrisalova et al., 2019). Each patient was asked to carefully demonstrate manipulation with a placebo inhaler. Next, patients treated with combination therapy with two or more different types of inhalers demonstrated their use of each type. All types of inhalers authorized and used in the treatment of COPD at the time of the study were evaluated (Czech Multicenter Research Database of COPD, 2022). Then, adherence to each of the individual types of inhalers was expressed as errors at any of the five, clearly predefined steps (total score from 0 to 5; application without any error at any steps = 0) for each type of inhaler. A-ApplT was evaluated and recorded by a nurse. Both types of adherence were evaluated under the direct supervision (in the same room) of a respiratory physician. Before the start of the project, respiratory nurses and physicians were systematically educated/trained in several phases. Moreover, all medical staff demonstrated full-text access to all educational documents. In addition, during the research, all participating centers were inspected by the study coordinators (Vladimir Koblizek, Magda Vytrisalova).

### Statistical Analysis

Patients with information on at least one adherence parameter were included in the analysis. Statistical differences in continuous variables among groups were tested with the Mann–Whitney *U* test. Relationships between two continuous parameters were analyzed with Spearman’s correlation. Also, relationships between two categorical parameters were analyzed with Fisher’s exact test. Predictors of a higher ©MMAS-4 score (> 0; lower adherence) and errors during inhalation (> 0) were analyzed using logistic regression. Parameters with a statistically significant relationship with ©MMAS-4 score/errors during inhalation (Mann–Whitney *U* test, Spearman’s correlation, Fisher’s exact test) were entered in a univariate regression model used in the first step. Then, statistically significant parameters were entered into a multivariate model. Data were analyzed using IBM SPSS Statistics 24.0.0.0. The level of significance was preset to *α* = 0.05.

## Results

Thirteen centers measuring adherence recruited 546 participants. This represents 69.6% of all patients (N = 784) included in the CMRD of COPD. Participants’ sociodemographic and main clinical characteristics are summarized in Supplementary Table S1 **.** The majority of COPD patients (88%) used a combination of two or more inhalation systems. Further details on the types of inhalation systems and their combinations used by patients have been published previously (Vytrisalova et al., 2019).

### Overall Adherence

**FIGURE 1:**
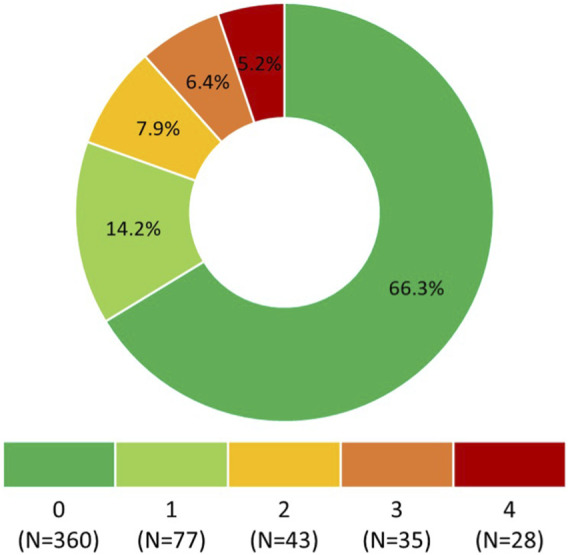
Morisky Medication Adherence Scale (©MMAS-4) score. 0 points means the highest adherence, 4 points means the lowest adherence. Morisky Widget™, MMAS-4™ (Morisky Medication Adherence Scale™), Morisky Medication Adherence Protocol™, Morisky Medication Adherence Scale, content, name, and trademarks are protected by U.S. and International Trademark and Copyright laws. Permission for use of the scale and its coding is required. A license agreement is available from Donald E. Morisky, ScD, ScM, MSPH, 14725 NE 20th St Bellevue, WA 98007, United States; dmorisky@gmail.com.

Responses to the questionnaire items are summarized in Table 1. According to Morisky Medication Adherence Scale (©MMAS-4), 360 (66.3%) participants self-reported optimal OA, i.e., 0 points (Figure 1).

**TABLE 1:** Overall adherence based on Morisky Medication Adherence Scale (©MMAS-4).

Item	N (%)
1. Do you ever forget to take your COPD medicine?	130 (23.9)
2. Do you ever have problems remembering to take your COPD medication?	76 (14.0)
3. When you feel better, do you sometimes stop taking your COPD medicine?	90 (16.6)
4. Sometimes if you feel worse when you take your COPD medicine, do you stop taking it?	84 (15.5)
**Total score**	
mean ± SD; median (5–95% quantile)	0.7 ± 1.17; 0.0 (0.0; 4.0)

N–the number (and relative %) of patients responding: “yes.”

Morisky Widget™, MMAS-4™ (Morisky Medication Adherence Scale™), Morisky Medication Adherence Protocol™, Morisky Medication Adherence Scale, content, name, and trademarks are protected by U.S. and International Trademark and Copyright laws. Permission for use of the scale and its coding is required. A license agreement is available from Donald E. Morisky, ScD, ScM, MSPH, 14725 NE 20th St Bellevue, WA 98007, United States; dmorisky@gmail.com.

The ©MMAS-4 score (poorer OA) correlated positively with symptoms of depression measured by the Beck Scale (r = 0.123; *p* = 0.038) and the number of exacerbations treated at home (r = 0.132; *p* = 0.002). The score correlated negatively with years of education (r = −0.128; *p* = 0.004), the number of inhalers (r = −0.241; *p* < 0.001), and the intensity of upper respiratory tract symptoms (Sino-Nasal Outcome Test, SNOT-22) (r = −0.147; *p* = 0.001). Poorer OA was found in patients living alone (*p* = 0.050) and in those suffering from anemia, depression, peptic ulcer disease (*p* < 0.001), or a malignant tumor (*p* = 0.001). A statistically significant difference was found in the score between groups according to smoking status (*p* = 0.001; ex-smokers demonstrated a higher tendency to report optimal OA) and between groups according to mouthwash after application of inhaled corticosteroids (ICS) (*p* < 0.001; patients always rinsing their mouth tended to report optimal adherence). Patients without flu vaccination during the previous year showed poorer OA (*p* = 0.007).

**Multivariate logistic regression** showed peptic ulcer disease, anemia, and depression independently associated with poorer OA (higher ©MMAS-4 score). On the contrary, flu vaccination and the higher degree of education were associated with better OA. More details are available in Table 2 **.**

**TABLE 2:** Independent predictors of suboptimal overall adherence (Morisky Medication Adherence Scale ©MMAS-4 > 0).

		Univariate logistic regression		Multivariate logistic regression*	
		OR (95% CI)	*p*	OR (95% CI)	*p*
Beck Depression Inventory		1.08 (1.01–1.15)	**0.029**		
Education level (years spent in a pregraduate school)		0.88 (0.81–0.95)	**0.001**	0.90 (0.82–0.98)	**0.013**
Living alone		1.52 (1.00–2.30)	**0.049**		
Regular mouthwash after ICS inhalation	*Always*	References category			
	Sometimes	3.45 (2.00–5.96)	**< 0.001**		
	Absence of ICS	2.64 (1.67–4.18)	**< 0.001**		
	Never	3.03 (1.24–7.39)	**0.015**		
Smoking status	*Current smoker*	References category			
	Ex-smoker	0.48 (0.31–0.76)	**0.001**		
	Non-smoker	0.96 (0.50–1.85)	0.905		
Malignancy		2.28 (1.39–3.76)	**0.001**		
Anemia		5.82 (3.15–10.76)	**< 0.001**	3.79 (1.76–8.16)	**< 0.001**
Depression		3.33 (2.15–5.17)	**< 0.001**	3.01 (1.67–5.42)	**< 0.001**
Peptic ulcer		2.81 (1.83–4.32)	**< 0.001**	2.70 (1.57–4.64)	**< 0.001**
Flu-vaccination		0.62 (0.40–0.94)	**0.026**	0.55 (0.32–0.94)	**0.029**
Types of inhalers in one patient		0.51 (0.39–0.67)	**< 0.001**		
FEV_1_ (% pred)		1.03 (1.01–1.05)	**< 0.001**	1.03 (1.01–1.05)	**0.002**
FEV_1_/FVC (%)		4.76 (1.14–19.87)	**0.032**		
RV (% pred)		0.99 (0.99–1.00)	**< 0.001**		
TLC (% pred)		1.00 (0.98–1.00)	**0.003**		
TL_CO_ (% pred)		1.02 (1.01–1.04)	**< 0.001**		
6MWD (m)		1.00 (1.00–1.01)	**< 0.001**	1.00 (1.00–1.00)	**0.009**

*Statistically significant parameters from the univariate logistic regression were the entry into a multivariate model. The table only shows the statistically significant parameters in multivariate analysis.

Statistically significant results are provided in bold.

Abbreviations: ICS, inhalation corticosteroids; FEV_1_, postbronchodilator forced expiratory volume in 1 s; FVC, forced vital capacity; RV, residual volume; TLC, total lung capacity; TL_CO_, transfer factor of the lung for carbon monoxide; 6MWD, the 6-min walking distance.

### Adherence to Application Technique (Five Steps Assessment)

The assessment of A-ApplT revealed that 164 (32%) participants adhered properly to each of the five steps. The highest rate of failure was observed in steps No. 3 (failure to breathe out completely in one breath immediately before inhalation of the drug) and No. 4 (actual inhalation). The complete results of A-ApplT considering the different types of inhalers used have been published elsewhere (Vytrisalova et al., 2019).

The participants using a higher number of inhalers showed more errors (r = 0.359; *p* < 0.001) as did the participants with anemia and peptic ulcer disease history (*p* = 0.001, and *p* = 0.033, respectively). A statistically significant difference was found in the number of errors between groups according to mouthwash after application of ICS (*p* < 0.001; patients always rinsing their mouth tended to inhale without any error).

**The multivariate analysis** identified the absence of regular post-ICS mouthwash and higher COPD Assessment Test (CAT) as independent risk factors for an incorrect inhalation technique. However, higher degree of education was associated with a significantly better inhalation technique adherence. More details are available in Table 3 **.**

**TABLE 3:** Independent predictors of errors during inhalation (Five Steps Assessment >0).

		Univariate logistic regression		Multivariate logistic regression*	
		OR (95% CI)	*p*	OR (95% CI)	*p*
Education level (years spent in pregraduate school)		0.89 (0.83–0.96)	**0.002**	0.92 (0.85–0.99)	**0.033**
Total exacerbation rate/previous year		1.16 (1.02–1.31)	**0.023**		
COPD symptoms (CAT)		1.03 (1.00–1.051)	**0.049**	1.03 (1.00–1.06)	**0.048**
Regular mouthwash after inhalation of ICS	*Always*	References category		References category	
	Sometimes	3.57 (1.90–6.68)	**< 0.001**	2.63 (1.28–5.40)	**0.008**
	Absence of ICS	1.30 (0.86–1.97)	0.208	1.20 (0.73–1.95)	0.475
	Never	15.19 (2.01–115.04)	**0.008**	10.75 (1.39–83.15)	**0.023**
Anemia		3.97 (1.66–9.48)	**0.002**		
Peptic ulcer		1.76 (1.06–2.92)	**0.029**		
IC/TLC (%)		0.99 (0.98–1.00)	**0.004**		
6MWD (m)		1.00 (1.00–1.00)	**0.008**	1.00 (1.00–1.00)	**0.030**

*Statistically significant parameters from the univariate logistic regression were the entry into a multivariate model. The table only shows the statistically significant parameters in multivariate analysis.

Statistically significant results are provided in bold.

Abbreviations: CAT, COPD Assessment Test; COPD, chronic obstructive pulmonary disease; ICS, inhaled corticosteroids; FEV_1_, postbronchodilator forced expiratory volume in 1 s; IC, inspiratory capacity; TLC, total lung capacity; 6MWD, the 6-min walking distance.

Lower values of inspiratory capacity to total lung capacity ratio (IC/TLC) were associated with incorrect technique both in steps No. 3 and No. 4. More frequent exacerbations were observed among participants with a tendency to make errors in step No. 3 (Table 4).

**TABLE 4:** Associations between critical errors in application technique (Five Steps Assessment) and pulmonary functions and rate of exacerbations.

Functional parameter	Error in inhalation technique		*p*
	Error in step No. 3		
	No (N = 262)	Yes (N = 261)	
**Post-bronchodilator FEV**_**1**_ **(% of predictive values)** median (5–95% quantile)	45.93 (26.24–59.80)	45.62 (24.87–60.49)	0.996
**FEV**_**1**_ **/FVC (%)** median (5–95% quantile)	0.53 (0.33–0.74)	0.50 (0.34–0.72)	0.128
**IC/TLC (%)** median (5–95% quantile)	32.00 (17.00–78.44)	28.00 (15.00–95.16)	**0.004**
**Total number of exacerbations** median (5–95% quantile)	0.00 (0.00–4.00)	1.00 (0.00–4.00)	**0.013**
	**Error in step No. 4**		
	No (N = 323)	Yes (N = 200)	
**Post-bronchodilator FEV**_**1**_ **(% of predictive values)** median (5–95% quantile)	46.12 (25.15–60.03)	44.74 (25.45–60.04)	0.433
**FEV**_**1**_ **/FVC (%)** median (5–95% quantile)	0.53 (0.35–0.71)	0.49 (0.32–0.74)	**0.011**
**IC/TLC (%)** median (5–95% quantile)	32.00 (15.00–95.00)	29.00 (18.00–50.00)	**0.003**
**Total number of exacerbations** median (5–95% quantile)	1.00 (0.00–4.00)	1.00 (0.00–4.50)	0.576

Statistically significant results are provided in bold.

FEV_1_, postbronchodilator forced expiratory volume in 1 s; FVC, forced vital capacity; TLC, total lung capacity; IC, inspiratory capacity.

### Relationship Between Overall Adherence and Adherence to Application Technique

**FIGURE 2:**
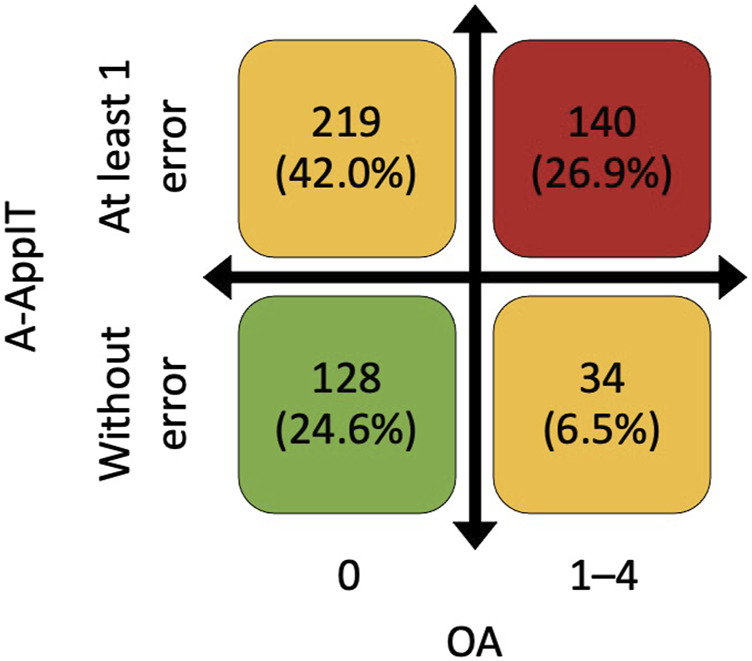
Combination of overall adherence (OA) based on Morisky Medication Adherence Scale (©MMAS-4) and adherence to application technique (A-ApplT) based on Five Steps Assessment. OA—0 points means the highest adherence, and 4 points means the lowest adherence. A-ApplT–numbers of errors in all inhalation systems used.

OA did not correlate with A-ApplT (r = 0.097; *p* = 0.066). Poorer OA was associated with better lung function i. e. ↑ postbronchodilator forced expiratory volume in 1 s (FEV_1_), ↑FEV_1_/forced vital capacity (FVC), ↓residual volume (RV), ↓TLC, ↑transfer factor of the lung for carbon monoxide (TL_CO_), and better exercise tolerance (↑ the 6-min walking distance, 6MWD). By contrast, incorrect application technique (A-ApplT) was associated with poorer lung function (↓FEV_1_, ↓IC/TLC) and lower exercise tolerance (↓6MWD). More details are available in Table 5. A comparison of simultaneously ascertained OA and A-ApplT revealed that 42% of patients showed optimal OA but poor A-ApplT, and 27% of patients showed poor both types of adherence (OA and A-ApplT). On the contrary, 25% of our cohort demonstrated optimal both types of adherence. At last, 6% of patients reported poor OA, but their inhalation technique was observed to be flawless (Figure 2; Table 6).

**TABLE 5:** Correlations between Morisky Medication Adherence Scale (©MMAS-4) score and the number of errors in application technique (Five Steps Assessment) with pulmonary functions (r; *p*-value).

Parameter	©MMAS-4 score (overall adherence)	No of errors in application technique (adherence to application technique)
FEV_1_ (% pred)	**0.156 (< 0.001)**	**-0.143 (0.007)**
FEV_1_/FVC (%)	**0.147 (< 0.001)**	-0.080 (0.130)
RV (% pred)	**-0.211 (< 0.001)**	0.036 (0.538)
TLC (% pred)	**-0.180 (< 0.001)**	0.040 (0.495)
IC/TLC (%)	0.034 (0.515)	**-0.136 (0.036)**
TL_CO_ (% pred)	**0.260 (< 0.001)**	-0.108 (0.065)
6MWD (m)	**0.250 (< 0.001)**	**-0.140 (0.012)**

Statistically significant results are provided in bold.

Abbreviation: r - Spearman's correlation coefficient; ©MMAS-4, Morisky Medication Adherence Scale 4; FEV_1_, postbronchodilator forced expiratory volume in 1 s; FVC, forced vital capacity; RV, residual volume; TLC, total lung capacity; IC, inspiratory capacity; TL_CO_, transfer factor of the lung for carbon monoxide; 6MWD, the 6-min walking distance.

**TABLE 6:** Combination of overall adherence (OA) based on Morisky Medication Adherence Scale (©MMAS-4) and adherence to application technique (A-ApplT) based on Five Steps Assessment.

		Valid N^a^	N (%)
Patients with known information about at least one parameter (OA and/or A-ApplT)			
OA (©MMAS-4)	0	(N = 543)	360 (66.3%)
	1–4		183 (33.7%)
A-ApplT	Without error	(N = 523)	164 (31.4%)
	At least 1 error		359 (68.6%)
Patients with known information about both parameters (OA and A-ApplT)			
OA (©MMAS-4)	0	(N = 521)	347 (66.6%)
	1–4		174 (33.4%)
A-ApplT	Without error	(N = 521)	162 (31.1%)
	At least 1 error		359 (68.9%)
Combination	©MMAS-4 = 0 & Errors = 0	(N = 521)	128 (24.6%)
	©MMAS-4 >0 & Errors = 0		34 (6.5%)
	©MMAS-4 = 0 & Errors >0		219 (42.0%)
	©MMAS-4 >0 & Errors >0		140 (26.9%)

a Patients with known information.

A-ApplT–adherence to application technique; errors - errors in application technique (Five Steps Assessment); ©MMAS-4, Morisky Medication Adherence Scale 4; OA, overall adherence.

## Discussion

The present study provides robust data from baseline evaluation of medication adherence within a large observational multicenter database of COPD subjects in the Czech Republic. Based on Morisky Medication Adherence Scale (©MMAS-4), two thirds of our patients self-reported optimal OA. However, optimal adherence to the application technique (A-ApplT) evaluated by the Five Steps Assessment was observed in one third of patients only. Although OA did not correlate with A-ApplT, both aspects of adherence to inhalation therapy were, usually inversely, interrelated, with regards to clinical and functional parameters. Thus far, this is the largest study simultaneously measuring and, at the same time, exploring the association between quantitative adherence (OA to inhaled treatment), qualitative adherence (actual ability to inhale correctly), and other parameters in patients with non-mild COPD.

### Overall Adherence

Studies of adherence use various types of assessments (e.g., questionnaires, Likert scales, pharmacy claims, and dose count). To provide relevant discussion, we compared our results to those of studies using the same OA assessment (©MMAS-4) and involving patients with COPD only. When discussing the results concerning associated factors, we also included research measuring OA with other methods.

A study in Hungary by Agh et al. (Agh et al., 2011) regarded patients scoring 3 and 4 on ©MMAS-4 as completely or almost completely adherent (58.2%). If we applied the same approach, more than 80% of our participants would be found adherent (we used reverse scoring). Jarab et al. (Jarab and Mukattash, 2019) used the same ©MMAS-4 scoring but presented different results (38.3% of COPD patients with optimal OA). Affected by more severe COPD according to postbronchodilator forced expiratory volume in 1 s (FEV_1_), our participants might have been more motivated to adhere to the recommended treatment; perhaps, their motivation was even more increased by their inclusion in a large multicenter research project that required examination of a variety of conditions. Furthermore, higher reported adherence might have also been associated with the duration of treatment that was more than six years in our cohort and a higher degree of education that might be linked to better awareness in the area of inhalation adherence. Next, social desirability bias can also play a role because participants in our study were interviewed face-to-face by their health care providers, whereas patients in Jarab et al. were interviewed by a member of the research team (Jarab and Mukattash, 2019), and Agh et al. used self-reported postal questionnaires (Agh et al., 2011). A similarly high level of OA, as in our cohort, was observed by Contoli et al. (mean ©MMAS-4 total score at enrolment was 3.4 ± 0.9, i.e., comparable result to our 0.7 ± 1.17, as they used reverse scoring) (Contoli et al., 2019). According to the review by Restrepo et al., an average of 40–60% of COPD patients adheres to their prescribed regimen (Restrepo et al., 2008).

Similarly to other authors (Bourbeau and Bartlett, 2008; Agh et al., 2011; Jarab and Mukattash, 2019), we identified forgetting to use medication to be the most frequent problem. Also, we observed intentional dose reduction when feeling well (more than 15%); in the study by George et al., this was the most common cause of nonadherence (George et al., 2005).

In our study, lower OA was reported by current smokers. The correlation was not verified in multivariate analysis but corresponds with the results published by Agh et al. and Darba et al. (Agh et al., 2011; Darba et al., 2015). On the contrary, an international LASSYC study in Latin America reported poor adherence to be associated with shorter smoking history (de Oca et al., 2017).

Our research consistently shows that functionally more affected patients have a better (higher) OA, and this was demonstrated clearly in three functional parameters: 1) degree of bronchial obstruction–FEV_1_, 2) severity of pulmonary hyperinflation–residual volume (RV), total lung capacity (TLC), and 3) tolerance of physical activity—the 6-min walking distance (6MWD). The same trend was identified by Duarte-de-Araújo et al. (Duarte-de-Araújo et al., 2020). Furthermore, the assumption exists that more types of inhalers are used by clinically more affected patients. Patients with more severe disease are likely to be better adapted to their condition, e.g., since better informed, they can perceive their treatment better. A negative correlation has been reported for the relationship between adherence and the number of exacerbations (Chrystyn et al., 2014; Leiva-Fernandez et al., 2014; Darba et al., 2015). Our analyses confirmed the same in the field of moderate exacerbations.

The relationship between OA and comorbidities has previously been studied. Darba et al. (Darba et al., 2015) reported a relationship between adherence and neurological and cardiovascular comorbidities. In our study, peptic ulcer disease and anemia history were independent risk factors for lower OA. The incidence of peptic ulcer is significantly higher in alcoholics, or current smokers, than in abstainers. Therefore, the possibility exists that patients with peptic ulcer exhibit poorer adherence to treatment due to various mental cognitive impairments associated with alcohol abuse (Lee et al., 2017). Furthermore, increasing evidence exists that anemia leads to chronic impairment of mental and cognitive functions, and these could also present as lower adherence to treatment due to forgetfulness and reduced ability to perform inhalation without error (Korkmaz et al., 2015; Dlugaj et al., 2016). While this not been verified in a multivariate analysis, we found lower adherence to be related to both personal history of depression and a higher Beck Scale score. Other authors also showed a relationship between depression and OA (Bourbeau and Bartlett, 2008; Rogliani et al., 2017), where depression was associated with lower adherence.

Research on the association between flu vaccination and the OA to inhaled medication in COPD patients is lacking. Our results suggest that influenza vaccination might be more frequent in highly motivated patients as well as in patients with good (higher) OA.

### Adherence to Application Technique

Similar to other authors (Rootmensen et al., 2010; Laube et al., 2011; Hammerlein et al., 2011; Arora et al., 2014; Pothirat et al., 2015; Dudvarski et al., 2016; Bartolo et al., 2017), we previously showed steps No. 3 (breathing out completely before inhaling) and No. 4 (inhaling correctly) to be the most problematic. Further, we focused on the relationship between these critical errors and pulmonary functions and the rate of COPD exacerbations, i.e., the key parameters used to assess the patients’ actual course of disease in clinical practice. We found lower inspiratory capacity (IC)/TLC ratio, a sensitive predictor of static lung hyperinflation and overall COPD mortality (French et al., 2015), to be clearly associated with more errors during application. This is due to lower ability to exhale deeply/properly and breathe in strongly. Likewise, the occurrence of acute exacerbations is the strongest predictor of mortality in the COPD population (Schmidt et al., 2014). Exacerbated COPD individuals demonstrated progressively worsened static/dynamic lung hyperinflation (Global Strategy for the Diagnosis Management and Prevention of COPD, 2021). Therefore, it is not surprising that we found both these parameters to be significantly related to errors in steps No. 3 and No. 4.

In contrary, we found A-ApplT to be lower (worse) in participants using a higher number of inhalers. The literature on this is ambiguous, and while Rootmensen et al. showed the same, Pothirad et al. and Melani et al. did not (Rootmensen et al., 2010; Melani et al., 2011; Pothirat et al., 2015). We assume that patients with poorer clinical status (thus needing a combination treatment with various inhalers) are unable to perform correct inhalation even though they make an effort.

Next, we observed a significant relationship between A-ApplT and education. The link between lower educational levels and poorer A-ApplT is evident in the available literature (Melani et al., 2011; Arora et al., 2014; Pothirat et al., 2015; Bartolo et al., 2017). The evidence on the role of other sociodemographic variables is contradictory; some authors showed a significant relationship with age (Melani et al. and Arora et al.) or gender (Bartolo et al. and Pothirat et al.), while others did not (Hammerlein et al.) (Hammerlein et al., 2011; Melani et al., 2011; Arora et al., 2014; Pothirat et al., 2015; Bartolo et al., 2017).

Unlike bronchial asthma, inhalation corticosteroids (ICS) are not a mandatory treatment in COPD. However, ICS are recommended in frequently exacerbated COPD and in cases of clinical overlap between COPD and asthma (ACO) (Global Strategy for the Diagnosis Management and Prevention of COPD, 2021). Frequent exacerbators represent 30% of the CMRD population and, due to progressively impaired lung hyperinflation, are prone to errors in the ApplT. If these patients never rinse their mouths after ICS application, this may indicate lower cooperation with the inhalation system and a lower level of A-ApplT. The ACO patients form only 4% of our cohort; therefore, their impact in this matter is not substantial.

COPD Assessment Test (CAT) is a widely used multicomponent instrument to assess the real clinical impact of COPD in a specific patient (Jones et al., 2009; Global Strategy for the Diagnosis Management and Prevention of COPD, 2021). Multiple linear regression analysis of a large POPE Study cohort showed several variables significantly associated with higher CAT scores: the presence of depression, the higher number of previous exacerbations, decreased 6MWD, reduced FEV_1_(%), and higher modified Medical Research Council (mMRC) dyspnea scale (Miravitlles et al., 2019). It makes sense that more affected COPD patients with higher CAT score are more prone to faulty inhalation technique in our cohort.

### Relationship Between Overall Adherence and Adherence to Application Technique

The absence of a relationship between OA and A-ApplT could be surprising; however, both of the concepts exhibit their own background, typical correlates, and reasons (both intentional and unintentional), as also shown in our study. A-ApplT is traditionally understood as dexterity-based (daily life physical skill), although various other factors could further affect it. Whereas, OA is considered to be linked with patient behavior and motivation mainly reflecting their health beliefs and cognition (Azzi et al., 2017; George and Bender, 2019).

The relationship between these two different concepts of adherence has been studied by Azzi et al. in their research involving patients with asthma (Azzi et al., 2017). They found an association between inhaler technique and self-reported adherence in the previous 7 days. Their research differed from ours in methodology, terminology, and the cohort of patients involved; therefore comparison with our findings is difficult. The simultaneous analysis of both types of adherence was performed by Duarte de Araujo et al. (Duarte de Araujo et al., 2020), but they did not investigate the relationship between OA (detected by the Measure of Treatment Adherence questionnaire) and A-ApplT (assessed by using checklists of correct steps). Non-OA was reported by 47 (16.5%) patients, and a significant negative association was found between OA and CAT score and FEV_1_% (similarly to our file). Next, inhaler misuse was observed in only 113 (39.6%) patients, and it was not associated with CAT score, dyspnea, exacerbations nor FEV_1_% (we observed an association between better A-ApplT and better lung function). An interventional prospective study in Spain evaluated OA (dose counting) and correct inhalation technique (Leiva Fernandez et al., 2014). They found that adherence was favored by a lower number of exacerbations (similarly to our file) and a lower number of inhaler devices (contrary to our results). Also, A-ApplT was not analyzed in all patients, and detailed analysis of the relationship between OA and A-ApplT was not performed. The study was aimed at assessing the effectiveness of comprehensive education (Leiva Fernandez et al., 2014).

### Strengths

The strengths and limitations of our approach were discussed in detail in our previous publication (Vytrisalova et al., 2019). Here, we only briefly mention the main positives and negatives and add comments relevant to the present analysis. We consider the size and homogeneity of our cohort of consecutive patients with moderate to very severe COPD and completion of the assessments in routine clinical practice to be the main strengths of our study.

Thanks to the range of data collected in the CMRD of COPD, we tested a broad spectrum of factors as potential correlates. All factors were assessed using validated instruments, the majority of them used globally for monitoring in routine clinical practice.

### Limitations

The results could be affected by patient refusal to participate in the study due to lower motivation or poor health status. Next, completion of questionnaires is time-consuming and can also be bothersome for patients. Both instruments for depression assessment were sometimes refused, probably due to the stigmatization of psychiatric conditions.

Observational studies close to routine clinical practice, particularly those aiming to capture wide-ranging clinical evaluations, often face the problem of occasionally missing data. Likewise, our research suffers from some missing data, since the assessments within the CMRD of COPD are categorized into mandatory and recommended assessments, while the evaluation of adherence is rendered only a recommended one. When mandatory data were not available, the patient was excluded from the analysis (Novotna et al., 2014).

As mentioned in our previous publication, (Vytrisalova et al., 2019), the assessment of A-ApplT by investigators was subjective. A similar problem arises with the use of a self-reported questionnaire to measure OA. Patients often tend to overestimate their adherence or even intentionally speak untruth to as face-saving, and this could distort the results. This could also be one of the reasons for such a difference in rates between the two types of adherences observed.

Next, the novelty of our manuscript lies in the simultaneous measurement and intercomparison of OA and A-ApplT together with monitoring of a large number of sociodemographic, clinical, and functional parameters in non-mild COPD patients. The system for monitoring the A-ApplT (Five Steps Assessment) is also innovative; it is a universal tool for all types of inhalers, which is easy to use in routine clinical practice. Moreover, our study analyzed OA and A-ApplT parameters in the largest cohort of COPD patients to date.

It seems A-ApplT and OA are two separate yet partially overlapping constructs. Being aware of which type is measured in the studies and in clinical practice is necessary for the best use of results. Further research is needed to gain a better understanding of the relationship between these two domains of adherence, and their correlates in the area of respiratory medicine.

## Data Availability

The data analyzed in this study is subject to the following licenses/restrictions: data of COPD project are stored in a database system, which was originally based on a modified version of TrialDB system. The system has been designed as a robust base for collection of a large amount of data in clinical trials and/or clinical registries, and it is fully customized to the structure of COPD project. The online application is accessible to users *via* the internet browser. The security of individual records within the registry is guaranteed *via* deidentified data collection. Each patient’s identity is replaced with a number (ID) which does not allow any backward identification of that person. The unequivocal identification of patient is only known to the attending physician or to an authorized health care professional. Requests to access these datasets should be directed to VK, vladimir.koblizek@fnhk.cz.

## Supplementary Material

The Supplementary Material for this article can be found online: https://www.frontiersin.org/articles/10.3389/fphar.2022.860270/full#supplementary-material
