# Supplementary material for: Self-Reported Overall Adherence and Correct Inhalation Technique Discordance in Chronic Obstructive Pulmonary Disease Population
Source: Front Pharmacol. 2022 Aug 12;13:860270. doi: 10.3389/fphar.2022.860270 (PMC9411979; doi:10.3389/fphar.2022.860270)
Supplement: Supplementary file 1 [file Table1.DOCX]

Supplementary Material

# Supplementary Table

**Supplementary Table 1** Characteristics of the study population with adherence assessment (N=546)

| **Basic demography** | |
| --- | --- |
| **Age** at entrance into the CMRD study in years  mean; median (5-95% quantile) | N=546  66.7; 67.0 (51.4-79.8) |
| **Duration of COPD** (from the first diagnosis to study enrollment) in years mean; median (5-95% quantile) | N=519  8.2; 6.6 (0.5-22.3) |
| **Men** (%) | 408 (74.7) |
| **Education level**: years spent in pre-graduate school  mean; median (5-95% quantile) | N=515  12.3;12.0 (9.0-18.0) |
| **Smoking status**  Ex-smokers (%)  Non-smokers (%)  Current smokers (%) | 386 (70.7)  56 (10.3)  104 (19.0) |
| **BMI** (kg/m^2^)  mean; median (5-95% quantile) | N=546  27.6; 27.3 (18.3-37.5) |
| **Medical characteristics** |  |
| **Moderate and severe exacerbations* during the last 12 months before enrollment** mean; median (5-95% quantile)  Total number (%) of patients who experienced at least one episode | N=546  1.3; 1.0 (0.0; 4.0)  296 (54.2%) |
|  | |
| COPD symptoms – **CAT score**  mean; median (5-95% quantile)  Upper respiratory tract symptoms – **SNOT-22 score**  mean; median (5-95% quantile) | N=541  15.3; 15.0 (3.0; 28.0)  N=496  17.4; 13.0 (1.0–47.0) |
| **Pulmonary functions** | |
| **FEV_1_** (% pred)  mean; median (5-95% quantile)  **FVC** (% pred)  mean; median (5-95% quantile)    **FEV_1_/FVC**  mean; median (5-95% quantile)  **RV** (% pred)  mean; median (5-95% quantile)  **TLC** (% pred)  mean; median (5-95% quantile)  **IC/TLC**  mean; median (5-95% quantile)    **TL_CO_** (% pred)  mean; median (5-95% quantile)  **6MWD** (m)  mean; median (5-95% quantile) | N=546  44.7; 45.7 (25.1-60.0)  N=546  68.6; 67.9 (39.8-100.7)  N=546  0.5; 0.5 (0.3-0.7)  N=463  185.9; 183.0 (99.0-291.0)  N=460  111.0; 110.0 (69.0-156.0)  N=373  36.7; 30.0 (17.0-79.0)  N=422  52.7; 50.5 (23.0-97.0)  N=473  325.8; 348.0 (110.0-530.0) |
| **Co-morbidities** | |
| Total number (%)  **Diabetes mellitus**  **Coronary artery disease**  **Cardiac failure**  **Atrial fibrillation**  **Hypertension**  **Malignant tumour**  **Osteoporosis**  **Anaemia**  **Depression**  **Peptic ulcer disease**  **Sleep apnoea syndrome** | 135 (24.7)  133 (24.4)  94 (17.2)  72 (13.2)  337 (61.7)  73 (13.4)  75 (13.7)  55 (10.1)  104 (19.0)  109 (20.0)  35 (6.4) |
| **Vaccination** | |
| **Influenza (during the last year)**  Total number (%) | 142 (26.4%) |
| **Pneumococcus (any time during the life)**  Total number (%) | 31 (5.8%) |
| **Depressive symptoms** | |
| **Zung** scale  mean; median (5-95% quantile)  **Beck** scale  mean; median (5-95% quantile) | N=274  49.9; 50.0 (32.0-68.0)  N=286  6.2; 6.0 (1.0-14.0) |

Abbreviations: BMI, body mass index; CAT, COPD Assessment Test; SNOT, Sino-Nasal Outcome Test (SNOT-22); FEV_1_, post-bronchodilator forced expiratory volume in 1 s; FVC, forced vital capacity; RV, residual volume; TLC, total lung capacity; IC, inspiratory capacity; TLCO, transfer factor of the [lung](https://en.wikipedia.org/wiki/Lung) for [carbon monoxide](https://en.wikipedia.org/wiki/Carbon_monoxide); 6MWD, the six-minute walking distance.

*deterioration of COPD symptoms which needs treatment with antibiotics and/or corticosteroids (oral or intravenous)

**
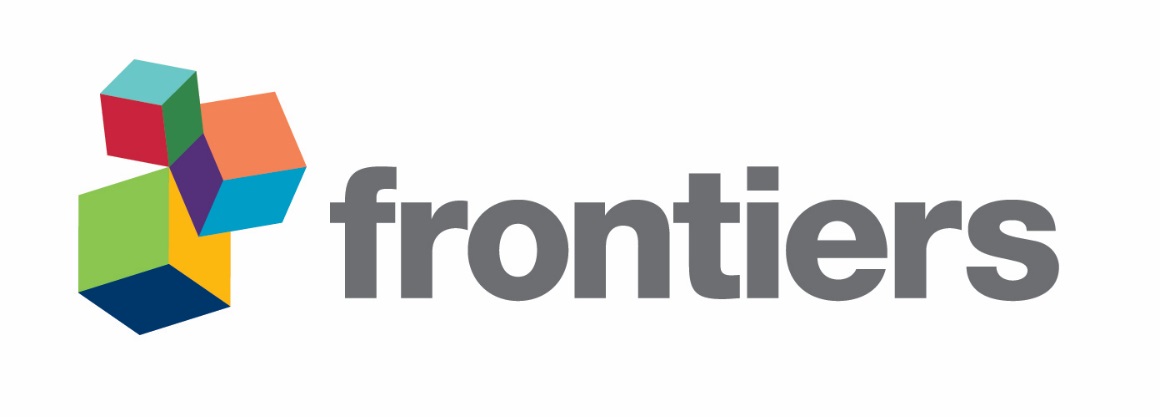
**
